# High expression of COMMD7 is an adverse prognostic factor in acute myeloid leukemia

**DOI:** 10.18632/aging.202901

**Published:** 2021-04-23

**Authors:** Kongfei Li, Lieguang Chen, Hua Zhang, Lu Wang, Keya Sha, Xiaohong Du, Daiyang Li, Zhongzheng Zheng, Renzhi Pei, Ying Lu, Hongyan Tong

**Affiliations:** 1 Department of Hematology, The First Affiliated Hospital, Zhejiang University School of Medicine, Hangzhou 310003, China; 2 Department of Hematology, People’s Hospital Affiliated to Ningbo University, Ningbo 315000, China; 3 Myelodysplastic Syndromes Diagnosis and Therapy Center, The First Affiliated Hospital, Zhejiang University School of Medicine, Hangzhou 310003, China; 4 Zhejiang Provincial Key Lab of Hematopoietic Malignancy, Zhejiang University, Hangzhou 310003, China; 5 Department of Hematology, Jinshan Hospital of Fudan University, Shanghai 201500, China; 6 Shanghai Tissuebank Biotechnology Co., Ltd, Shanghai 201318, China

**Keywords:** acute myeloid leukemia, COMMD7, The Cancer Genome Atlas, R packages

## Abstract

Acute myeloid leukemia (AML) is a frequent malignancy in adults worldwide; identifying preferable biomarkers has become one of the current challenges. Given that *COMMD7* has been reported associated with tumor progression in various human solid cancers but rarely reported in AML, herein, RNA sequencing data from TCGA and GTEx were obtained for analysis of *COMMD7* expression and differentially expressed gene (DEG). Furthermore, functional enrichment analysis of *COMMD7*-related DEGs was performed by GO/KEGG, GSEA, immune cell infiltration analysis, and protein-protein interaction (PPI) network. In addition, the clinical significance of *COMMD7* in AML was figured out by Kaplan-Meier Cox regression and prognostic nomogram model. R package was used to analyze incorporated studies. As a result, *COMMD7* was highly expressed in various malignancies, including AML, compared with normal samples. Moreover, high expression of *COMMD7* was associated with poor prognosis in 151 AML samples, as well as subgroups with age >60, *NPM1* mutation-positive, *FLT3* mutation-negative, and DNMT3A mutation-negative, et al. (*P* < 0.05). High *COMMD7* was an independent prognostic factor in Cox regression analysis; Age and cytogenetics risk were included in the nomogram prognostic model. Furthermore, a total of 529 DEGs were identified between the high- and the low- expression group, of which 92 genes were up-regulated and 437 genes were down-regulated. Collectively, high expression of *COMMD7* is a potential biomarker for adverse outcomes in AML. The DEGs and pathways recognized in the study provide a preliminary grasp of the underlying molecular mechanisms of AML carcinogenesis and progression.

## INTRODUCTION

Acute myeloid leukemia (AML) is an aggressive malignant tumor characterized by high heterogeneity, variable prognosis, and high mortality. The principal factors in risk stratification and treatment options are currently composed of cytogenetic and molecular abnormalities [1, 2]. However, the inherent concrete molecular mechanisms have not yet been exactly elucidated. The development of various targeted agents has facilitated individualized treatment for AML patients, thereby ameliorating complete remission (CR) rates and prolonging survival. Regrettably, the existing targeted drug monotherapy or combination therapy with traditional chemotherapy has not yet achieved the desired efficacy [3]. Thus, discerning novel biomarkers may contribute to better comprehending the molecular basis of AML, which may play an essential role in AML diagnosis, prognostic stratification, leukemia residual monitoring, treatment response prediction, as well as the possibility of targeted drug development.

COMM domain-containing protein 7 (*COMMD7*), a member of the COMMD family defined by the presence of a conserved and unique motif termed the copper metabolism gene MURR1 (COMM) domain, which is located on chromosome 20q11.21, has been reported associated with tumor progression in human solid cancers [4]. *COMMD7* is overexpressed in pancreatic ductal adenocarcinoma (PDAC) cells, associated with poor prognosis in PDAC patients. Inhibition of *COMMD7* gene in human PDAC cell lines induces antitumor effects under stress conditions, mediated in part by the *ERK1/2*-mediated *CyclinD 1*, *Bcl-2*, *Bax*, and *MMP-2* signaling pathways [5]. Another study revealed that *COMMD7*-overexpressed hepatocellular carcinoma (HCC) cells promoted the proliferation of naïve HCC cells [6]. On the other hand, *COMMD7* was identified as a novel *NEMO* interacting protein involved in *NF-κB* signaling termination [7], the incorrect regulation of which is known to be associated with a variety of tumors [5, 8]. One study demonstrated that *COMMD7* played a dual regulatory role in the *NF-κB* signaling pathway in HCC [9]. However, to date, the expression of *COMMD7* in AML and its prognostic value remain unclear.

Therefore, in this study, we aimed to ascertain the relationship between the expression level of *COMMD7* and the prognosis of AML by the following three steps: First of all, RNA sequencing (RNA-seq) data of AML samples from the cancer genome atlas (TCGA) and Genotype-Tissue Expression (GTEx) were acquired to analyze the expression of the core gene *COMMD7*. Subsequently, functional enrichment analysis of *COMMD7* was via GO, KEGG, GSEA, immune cell infiltration analysis, and protein-protein interaction (PPI) network. Besides, the clinical significance of *COMMD7* in AML was analyzed by Kaplan-Meier and Cox regression and nomogram prognostic model.

In this way, significantly altered genes and pathways would be screened out through gene enrichment analysis and subpathway enrichment analysis, the connection of which with *COMMD7* may play pivotal roles in the occurrence of AML.

## MATERIALS AND METHODS

### RNA-sequencing data and bioinformatics analysis

The pan-cancer RNA-seq data of TCGA and GTEx with toil processed uniformly were downloaded from UCSC XENA (https://xenabrowser.net/datapages/) [10–13]. Level 3 HTSeq-FPKM and HTSeq-Count data of the AML samples were obtained from the TCGA website (https://portal.gdc.cancer.gov/repository) for further analysis. This study was in full compliance with the published guidelines of TCGA and GTEx.

### Differentially expressed gene (DEG) analysis

The DESeq2 R package was adopted to compare expression data of low- and high-expression of *COMMD7* (cut-off value of 50%) in AML samples (HTseq-Count) to identify DEGs [14]. The top 10 DEGs were performed by heat map.

### Functional enrichment analysis

DEGs with the threshold for |logFC| >1.5 and padj <0.05 were applied for functional enrichment analysis. Gene Ontology (GO) functional analysis comprising cellular component (CC), molecular function (MF), and biological process (BP), as well as Kyoto Encyclopedia of Genes and Genomes (KEGG) pathway analysis, were implemented using the ClusteProfiler package in R [15].

### Gene set enrichment analysis (GSEA)

R package ClusteProfiler (3.14.3) was used for GSEA to elucidate the functional and pathway differences between the high- and low-expression groups of *COMMD7* [15]. The gene set was permutated 1,000 times for each analysis. Adjusted *P*-value < 0.05 and FDR q-value < 0.25 were considered to be statistically significant.

### Immune infiltration analysis by single-sample Gene Set Enrichment Analysis (ssGSEA)

Immune infiltration analysis of *COMMD7* was conducted by ssGSEA using GSVA package in R (3.6.3). A total of 24 types of infiltrating immune cells were obtained as previously described [16]. Spearman correction was used to analyze the correlation between *COMMD7* and the enrichment scores of 24 types of immune cells. Wilcoxon rank-sum test was used to analyze the enrichment scores of high- and low-*COMMD7* expression groups.

### PPI network

The PPI network of DEGs was predicted using the Search Tool for the Retrieval of Interacting Genes (STRING) database [17]. The interaction score threshold of 0.4 was set as the cut-off criterion. The PPI network was mapped using Cytoscape (version 3.7.1) [18], and the most significant modules in the PPI network were identified using MCODE (version 1.6.1) [19]. Selection criteria were as follows: MCODE scores >5, degree cut-off = 2, node score cut-off = 0.2, Max depth = 100, and k-score = 2. Metascape (https://metascape.org/gp/index.htm) was used to conduct the pathway and process enrichment analysis.

### Prognostic model generation and prediction

In order to individualize the prediction of overall survival (OS) and event-free survival (EFS) in AML patients, a nomogram was generated using the RMS R package (version 5.1-3), which included prominent clinical characteristics and calibration plots. The calibration curves were evaluated graphically by mapping the nomogram-predicted probabilities against the observed rates, and the 45°line represented the best predictive values. Concordance index (C-index) was used to determine the discrimination of the nomogram, and the bootstrap approach was used to calculate 1000 resamples. In addition, C-index and receiver operating characteristic (ROC) were used to analyze and compare the predictive accuracy of the nomogram and separate prognostic factors. All statistical tests were double-tailed with 0.05 as the statistical significance level.

### Statistical analysis

All statistical analyses and graphs were analyzed and displayed by R (3.6.2) [20]. The expression of *COMMD7* in unpaired samples was analyzed by Wilcoxon rank-sum test, with Wilcoxon signed-rank test used in paired samples. Kruskal-Wallis test, Wilcoxon signed-rank test, and logistic regression analysis were used to evaluate the relationship between clinical/cytogenetic characteristics and *COMMD7* expression. Cox regression analysis and Kaplan-Meier method were used to evaluate the prognostic factors. Multivariate Cox analysis was adopted to compare the impact of *COMMD7* expression on survival along with other clinical features. The median *COMMD7* expression was regarded as the cut-off value. In all tests, *P* value < 0.05 was considered statistically significant. Moreover, ROC analysis was performed on the pROC package to assess the effectiveness of the transcriptional expression of *COMMD7* in distinguishing AML from healthy samples. The computed area under the curve (AUC) value ranging from 0.5 to 1.0 indicated 50-100% discrimination ability.

## RESULTS

### *COMMD7* expression in pan-cancers and AML

**Figure 1:**
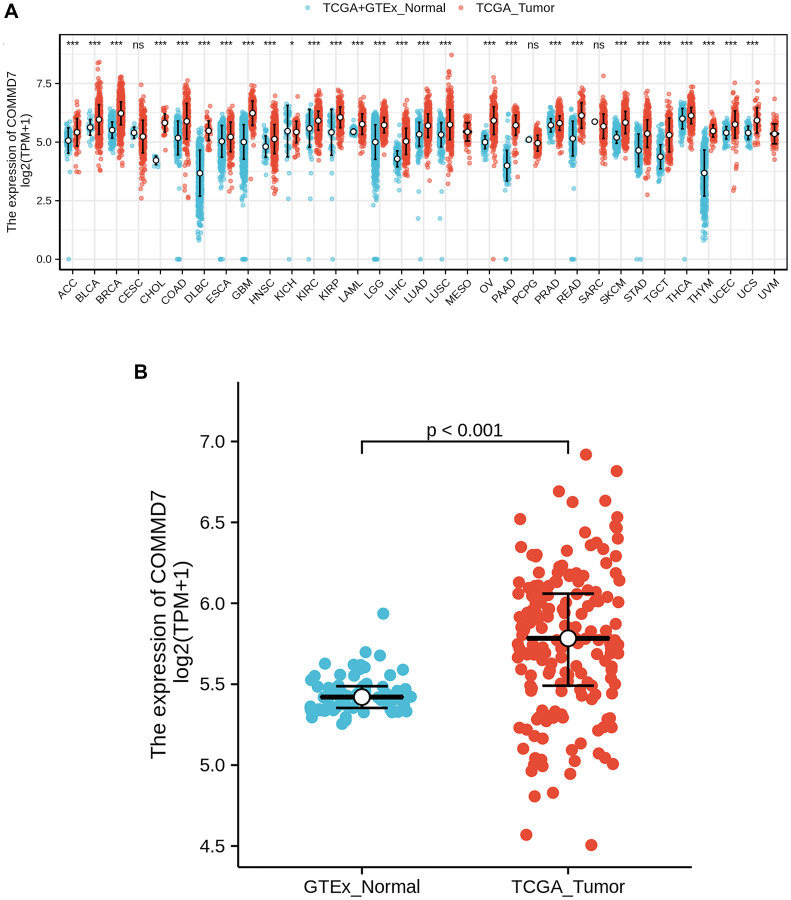
**The higher expression of *COMMD7* was showed in AML compared with normal samples.** (**A**) Expression level of *COMMD7* in paired normal and pan-cancer samples. (**B**) Expression level of *COMMD7* in paired normal and AML samples. Analysis between two groups: Wilcoxon Rank sum test; NS: *P* 0.05 or higher; ^*^P < 0.05; ^**^P < 0.01; ^***^P < 0.001.

RNA-seq data from UCSC XENA (https://xenabrowser.net/datapages/) was downloaded in TCGA and GTEx formats processed uniformly through the toil process. By comparing the expression of *COMMD7* normal samples in TCGA and GTEX database and corresponding tumor samples in TCGA database, *COMMD7* was found significantly high expressed in 28 types of cancer (Figure 1A), including acute myelogenous leukemia (LAML) (Figure 1B).

### Identification of DEGs in AML samples with low- and high-expressed *COMMD7*

**Figure 2:**
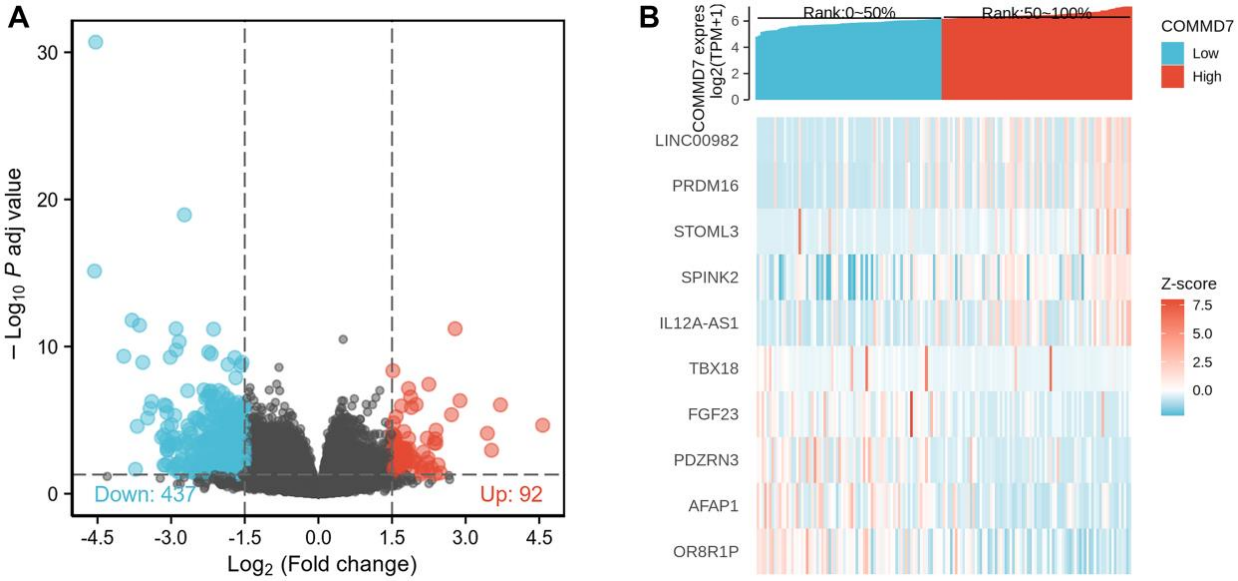
**A total of 529 DEGs were identified as being statistically significant between *COMMD7* high-expressed and low-expressed groups.** (**A**) Volcano plot of differentially expressed genes, including 92 up-regulated and 437 down-regulated genes. Normalized expression levels were shown in descending order from green to red. (**B**) Heat map of the 10 differentially expressed RNAs, including 5 up-regulated genes and 5 down-regulated genes. The X-axis represents the samples, while the Y-axis denotes the differentially expressed RNAs. Green and red tones represented down-regulated and up-regulated genes, respectively.

The high- and low-expression groups' gene expression profiles were analyzed for differences in the median mRNA expression. A total of 529 DEGs from gene expression RNA-seq-HTSeq-Counts, including 92 up-regulated and 437 down-regulated, were identified statistically significant between *COMMD7* high- and low-expressed groups (|log fold change (logFC)| > 1.5, *P* < 0.05) (Figure 2A). The top five up-regulated DEGs and top five down-regulated DEGs between *COMMD7* high- and low-expressed groups were illustrated by the heat map (Figure 2B).

### Functional enrichment analysis of DEGs

**Figure 3:**
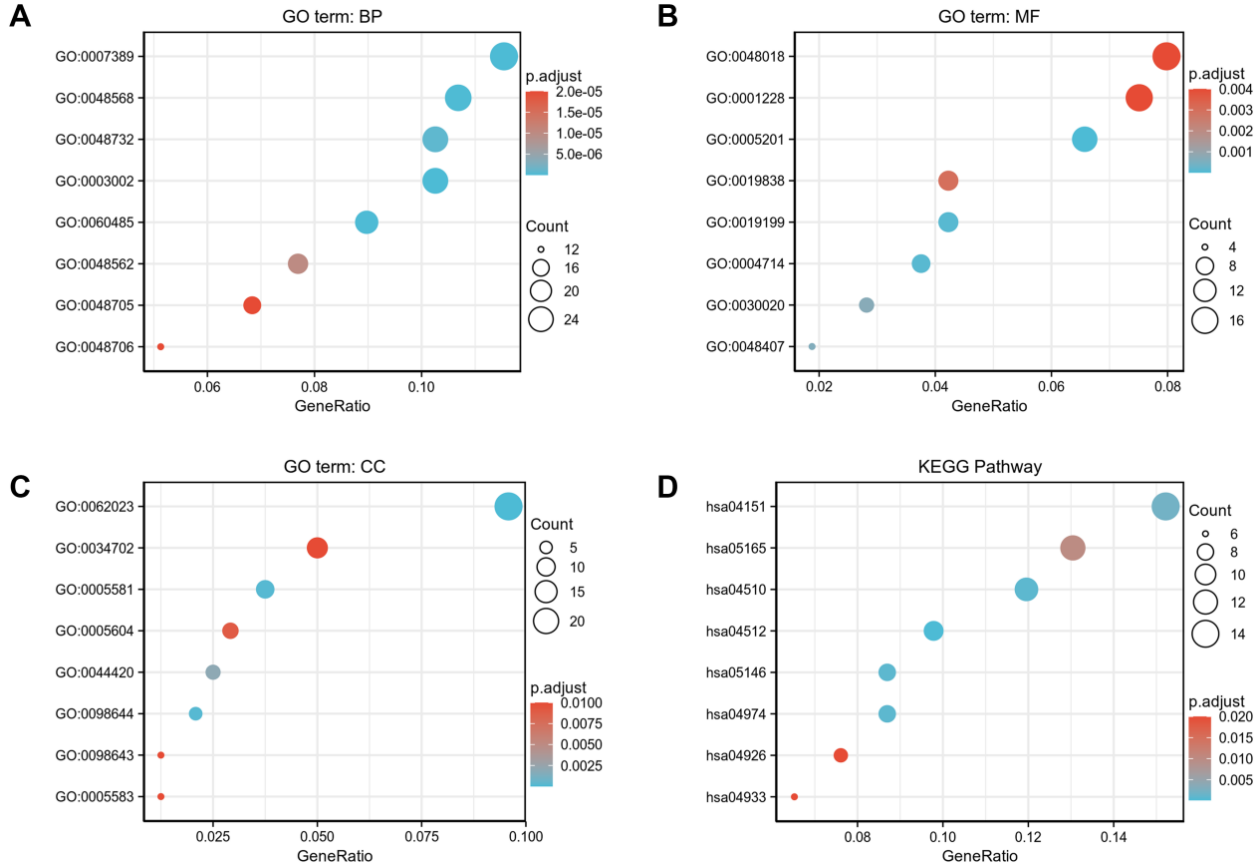
**GO/KEGG enrichment analysis of DEGs between high- and low- *COMMD7* expression in TCGA-LAML patients.** (**A**) Enriched GO terms in the “biological process” category; (**B**) Enriched GO terms in the “molecular function” category. (**C**) Enriched GO terms in the “cellular component” category; (**D**) KEGG pathway annotations. The X-axis represented the proportion of DEGs, and the Y-axis represented different categories. The different colors indicate different properties, and the different sizes represent the number of DEGs.

To better understand the functional implication of 529 DEGs between high- and low- expression of *COMMD7* in AML, GO and KEGG functional enrichment analysis was performed by clusterProfiler package (Supplementary Table 1, Figure 3). The association with the biological process (BP) included pattern specification process, regionalization, and mesenchyme development; cellular components (CC) included collagen-containing extracellular matrix, ion channel complex, and basement membrane; molecular function (MF) included receptor ligand activity, DNA-binding transcription activator activity/RNA polymerase II-specific, extracellular matrix structural constituent. KEGG included PI3K-Akt signaling pathway, focal adhesion, and *ECM*-receptor interaction.

**Figure 4:**
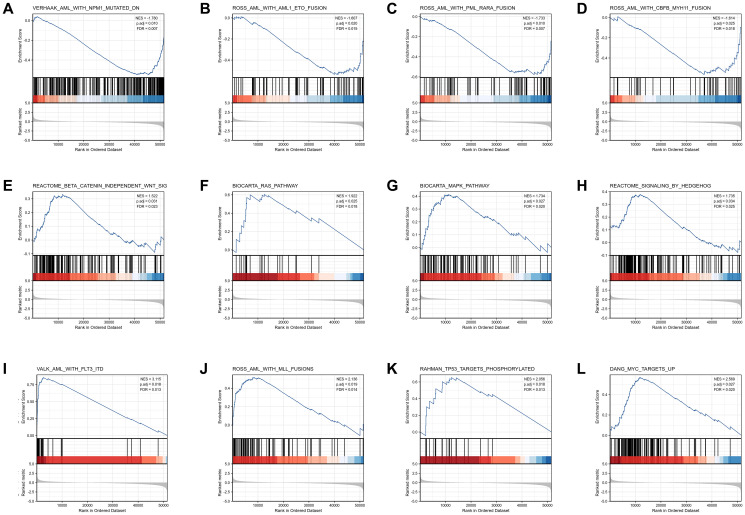
**Enrichment plots from the gene set enrichment analysis (GSEA).** (**A**–**L**) ES, enrichment score; NES, normalized ES; ADJ *P*-val, adjusted *P*-value.

GSEA analysis was conducted to gain further insight into the biologic pathways involved in AML with different *COMMD7* expression levels. GSEA was performed between low- and high-*COMMD7* expression datasets to identify critical signaling pathways involved in AML. Significant differences (FDR < 0.05, ADJ *P* < 0.05) were observed in the enrichment of MSigDB Collection (C2.all.v7.0.symbols.gmt) of these pathways (Supplementary Table 2 and Figure 4). Gene mutations or fusions with a good prognosis of AML, such as *PML-RARa* fusion, *NPM1* mutation, *AML-ETO* fusion, and *CBFB-MYH11* fusion, were enriched in *COMMD7* low- expression phenotype based on NES, with adjusted *P* value <0.05 and FDR value <0.05 (Figure 4A–4D). On the contrary, in the high expression of *COMMD7* phenotypes, factors with poor prognosis in AML, such as *FLT3-ITD* fusion and *MLL* fusion, presented significantly enriched (Figure 4I–4J). So did the pathways involved in AML and other tumor development, such as MAPK, RAS, Hedgehog, and Wnt pathways (Figure 4E–4H). Other genetic variants, such as phosphorylated TP53 targets and MYC targets, were also significantly enriched in such phenotype (Figure 4K–4L).

### Immune infiltration analysis in AML

**Figure 5:**
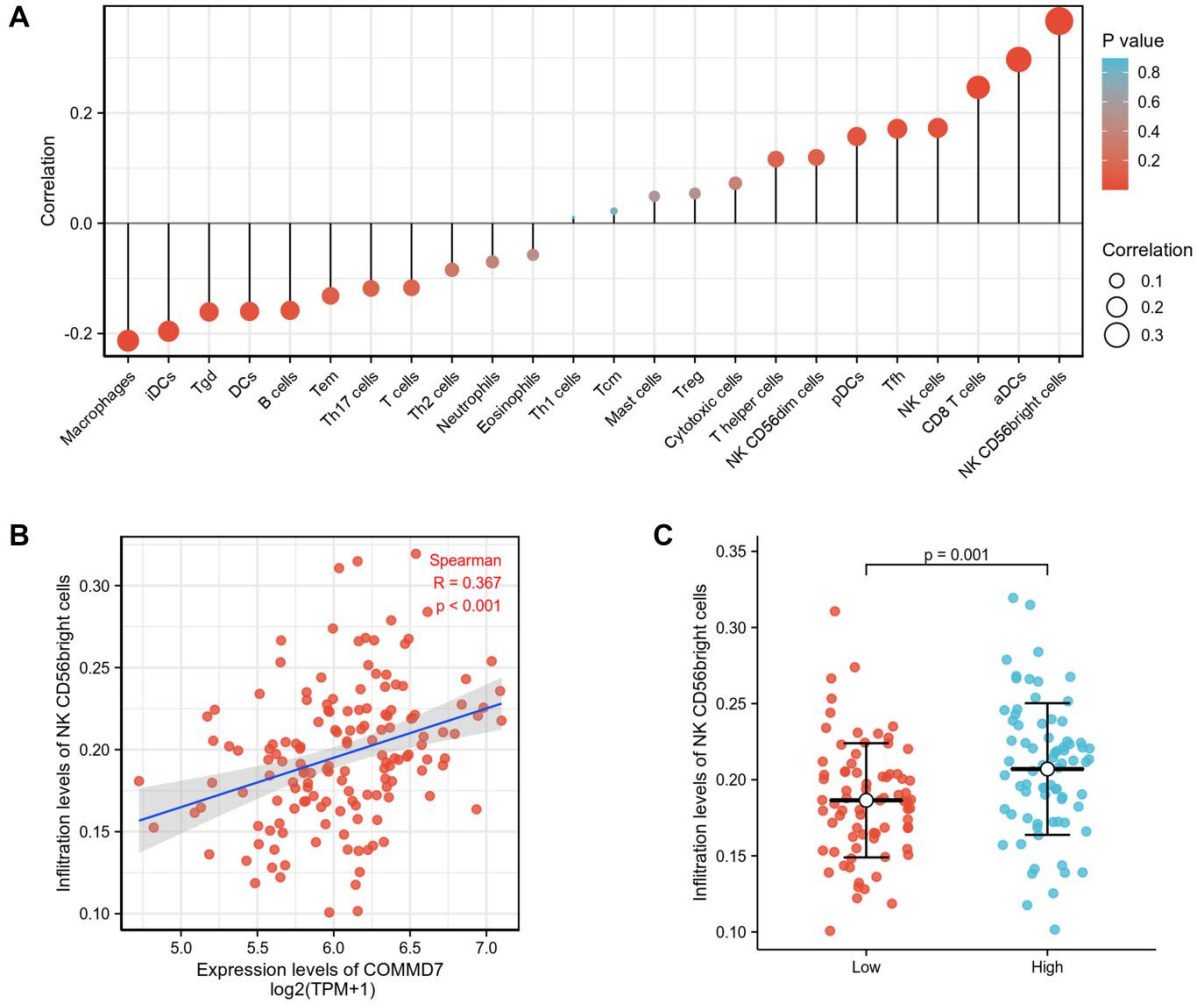
**The expression of *COMMD7* was associated with immune infiltration in the AML microenvironment.** (**A**), The forest plots showed a positive correlation between *COMMD7* and 13 immune cells, and a negative correlation between *COMMD7* and 11 immune cell subsets. The size of dots showed the absolute value of Spearman r. (**B**) Correlation between the relative enrichment score of NK CD56(bright) cells and the expression level (TPM) of *COMMD7*. (**C**) Infiltration of NK CD56(bright) cells between low- and high-*COMMD7* expressed.

Spearman correlation analysis showed that the expression level of *COMMD7* in the AML microenvironment was correlated with the immune cell infiltration level quantified by SSGSEA. Specifically, *COMMD7* was positively associated with NK CD56bright cells and active dendritic cells (aDCs) (Figure 5).

### PPI enrichment analysis in AML

**Figure 6:**
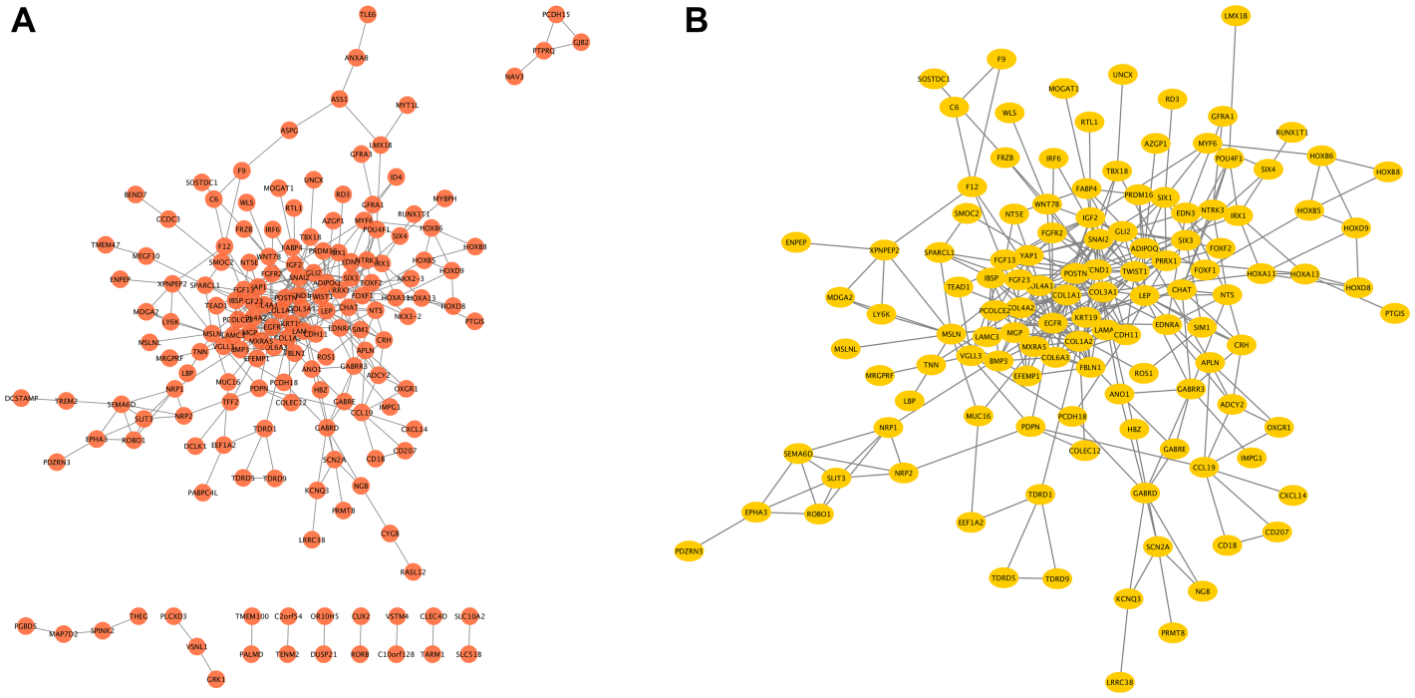
**The PPI network of *COMMD7*-related DEGs and the most significant module.** (**A**) The PPI network of DEGs was constructed using Cytoscape. (**B**) The most significant module was obtained from PPI network with 42 nodes and 150 edges.

The network of *COMMD7* and its potential co-expressed genes in *COMMD7*-related DEGs was constructed by STRING, with a threshold of 0.4 (Supplementary Table 3). A total of 529 DEGs were screened out ( |log fold change (logFC)| >1.5, *P* < 0.05). The PPI network with 238 nodes and 367 edges was displayed by Cytoscape-MCODE (Figure 6A). The most significant module with a MCODE score of 7.317 contained 42 nodes and 150 edges (Figure 6B). Meantime, Metascape-MCODE was used to identify densely connected PPI network components of *COMMD7*, shown in Supplementary Figure 1. The three best-scoring GO terms by *p*-value as the functional description of the corresponding components were shown in Supplementary Table 4.

### Association between *COMMD7* expression and clinical features and cytogenetic risks

The main clinical characteristics of AML in TCGA was shown in Table 1. A total of 151 cases (68 females and 83 males) were analyzed in this study, with an average age of 56.7 years. Among them, *COMMD7* expression was low in 76 (50.3%) AML patients and high in the remaining 75 (49.3%) cases. The median *COMMD7* expression (log2(TPM+1)), which is 5.783, was regarded as the cut-off value. Correlation analysis suggested that *COMMD7* expression was significantly correlated with cytogenetic risk and white blood cell (WBC) count (×10^9^/L) (*P* < 0.001). In addition, *COMMD7* expression was significantly associated with other factors including bone marrow (BM) (*P* = 0.007), peripheral blood (PB) blasts (%) (*P* = 0.005), FAB classification (*P* = 0.036), *FLT3* mutation (*P* = 0.004), *IDH1* R132 mutation (*P* = 0.046), and *NPM1* mutation (*P* = 0.014).

**Table 1:** Association between *COMMD7* expression and clinicopathologic features in AML samples from the TCGA database.

**Characters**	**level**	**Low expression of COMMD7**	**High expression of COMMD7**	***p***	**test**
**n**		76	75		
**Cytogenetic risk (%)**	**Favorable**	28 (36.8%)	3 (4.1%)	<0.001	
	**Intermediate**	29 (38.2%)	53 (72.6%)		
	**Poor**	19 (25.0%)	17 (23.3%)		
**FAB classifications (%)**	**M0**	7 (9.2%)	8 (10.8%)	0.036	exact
	**M1**	13 (17.1%)	22 (29.7%)		
	**M2**	18 (23.7%)	20 (27.0%)		
	**M3**	12 (15.8%)	3 (4.1%)		
	**M4**	19 (25.0%)	10 (13.5%)		
	**M5**	6 (7.9%)	9 (12.2%)		
	**M6**	0 (0.0%)	2 (2.7%)		
	**M7**	1 (1.3%)	0 (0.0%)		
**Cytogenetics (%)**	**+8**	6 (8.3%)	2 (3.2%)	<0.001	exact
	**Complex**	12 (16.7%)	12 (19.0%)		
	**del (5)**	0 (0.0%)	1 (1.6%)		
	**del (7)**	4 (5.6%)	2 (3.2%)		
	**inv (16)**	8 (11.1%)	0 (0.0%)		
	**Normal**	27 (37.5%)	42 (66.7%)		
	**t (15;17)**	8 (11.1%)	3 (4.8%)		
	**t (8;21)**	7 (9.7%)	0 (0.0%)		
	**t (9;11)**	0 (0.0%)	1 (1.6%)		
**Gender (%)**	**Female**	37 (48.7%)	31 (41.3%)	0.457	
	**Male**	39 (51.3%)	44 (58.7%)		
**Race (%)**	**Asian**	0 (0.0%)	1 (1.4%)	0.67	exact
	**Black or African American**	6 (8.0%)	7 (9.5%)		
	**White**	69 (92.0%)	66 (89.2%)		
***FLT3* mutation (%)**	**Negative**	14 (18.9%)	31 (42.5%)	0.004	
	**Positive**	60 (81.1%)	42 (57.5%)		
***IDH1R132* mutation (%)**	**Negative**	3 (4.0%)	10 (13.5%)	0.046	exact
	**Positive**	72 (96.0%)	64 (86.5%)		
***IDH1R140* mutation (%)**	**Negative**	8 (10.5%)	4 (5.5%)	0.369	exact
	**Positive**	68 (89.5%)	69 (94.5%)		
***IDH1R172* mutation (%)**	**Negative**	2 (2.6%)	0 (0.0%)	0.497	exact
	**Positive**	74 (97.4%)	73 (100.0%)		
***RAS* mutation (%)**	**Negative**	5 (6.6%)	3 (4.1%)	0.719	exact
	**Positive**	71 (93.4%)	71 (95.9%)		
***NPM1* mutation (%)**	**Negative**	10 (13.2%)	23 (31.1%)	0.014	
	**Positive**	66 (86.8%)	51 (68.9%)		
***DNMT3A* mutation (%)**	**Negative**	49 (89.1%)	43 (79.6%)	0.273	
	**Positive**	6 (10.9%)	11 (20.4%)		
***RUNX1* mutation (%)**	**Negative**	46 (83.6%)	51 (94.4%)	0.124	exact
	**Positive**	9 (16.4%)	3 (5.6%)		
**Age (median [IQR])**		55.50 [44.50,67.00]	58.00 [40.50,66.00]	0.816	nonnorm
**WBC count (x10^9/L) (median [IQR])**		11.00 [3.00,32.50]	35.00 [8.00,78.00]	<0.001	nonnorm
**BM blasts (%) (median [IQR])**		29.00 [5.00,55.75]	49.00 [14.00,71.50]	0.007	nonnorm
**PB blasts (%) (median [IQR])**		61.50 [41.75,79.50]	77.00 [57.50,86.00]	0.005	nonnorm

**Figure 7:**
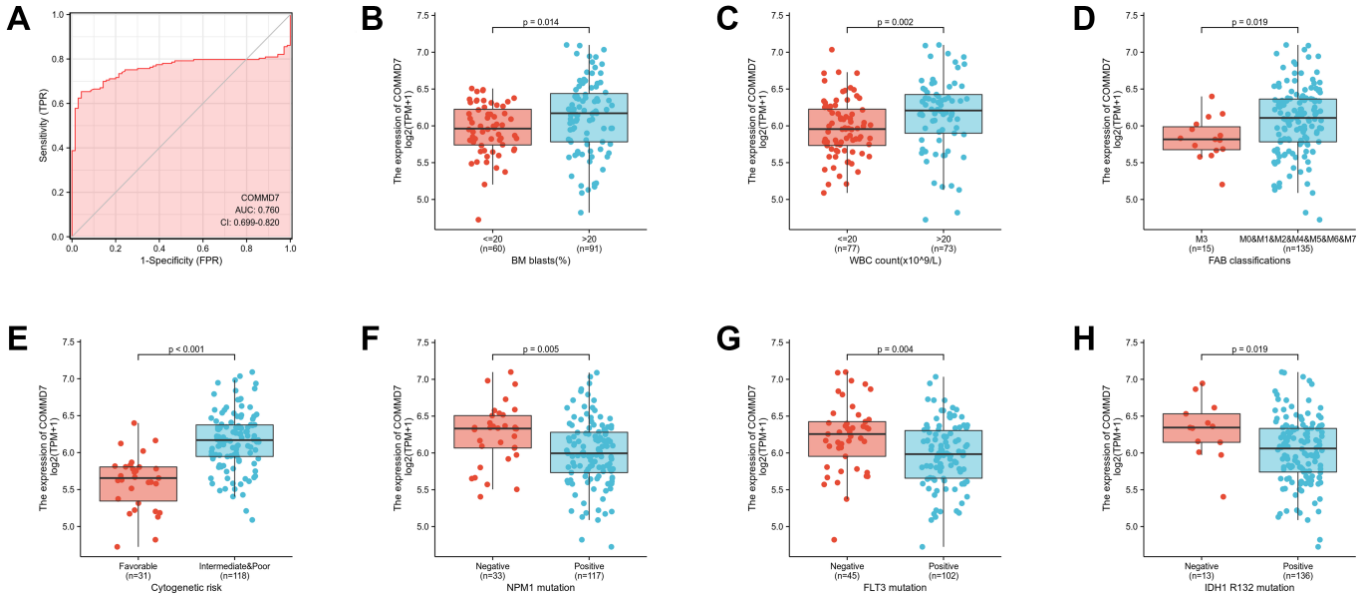
**Association between *COMMD7* expression and clinical features and cytogenetic risks.** (**A**) The diagnostic efficacy of *COMMD7* in acute myelogenous leukemia analyzed by ROC. (**B**–**H**) Association between *COMMD7* expression and BM blasts (20%), WBC counts (20 × 10^9^), FAB classification, cytogenetics risk, NPM1 mutation, FLT3 mutation, and IDH1 R132 mutation analyzed by using Wilcoxon Rank SUM test.

Logistic analysis was applied to further verify the relationship between AML clinicopathological factors and the *COMMD7* high-low dichotomy. As a result, high expression of *COMMD7* showed a significant positive correlation with high WBC count (>20 × 10^9^/L) (odds ratio [OR], 3.16; *P* < 0.001) and high PB blasts (>70%) (OR, 2.89; *P* = 0.002), whereas negatively correlated with *FLT3* mutation (OR, 0.32; *P* = 0.002) and *NPM1* mutation (OR, 0.34; *P* = 0.01) (Table 2). What is more, the potential value of *COMMD7* in differentiating AML patients from healthy individuals was examined by ROC curve analysis, with the AUC of 0.760, revealing that *COMMD7* had potential as a biomarker (Figure 7A). Besides, the Wilcoxon Rank SUM test was used to compare the expression of *COMMD7* in patients with different clinicopathological features. The result showed that *COMMD7* was significantly high-expressed in the patients with BM blasts (>20%; *P* = 0.014), WBC counts (>20 × 10^9^/L; *P* = 0.002), FAB classification (non-M3 type; *P* = 0.019), cytogenetics risk (intermediate/poor; *P* < 0.001), *NPM1* mutation (negative; *P* = 0.005), *FLT3* mutation (negative; *P* = 0.004), and *IDH1 R132* mutation (negative; *P* = 0.019) (Figure 7B–7H).

**Table 2:** The relationship between the clinicopathological factors of AML and *COMMD7* expression by using logistic analysis.

**Characteristics**	**Odds Ratio in *COMMD7* expression**	**Odds Ratio (OR)**	***P* value**
**WBC count(x10^9/L) (>20 vs. <=20)**	150	3.16(1.64–6.24)	<0.001
**PB blasts (%) (>70 vs. <=70)**	151	2.89(1.50–5.66)	0.002
**BM blasts (%) (>20 vs. <=20)**	151	1.91(0.99–3.74)	0.055
**Cytogenetic risk (Poor vs. Favorable&Intermediate)**	149	0.91(0.43–1.93)	0.807
***FLT3* mutation (Positive vs. Negative)**	147	0.32(0.15–0.65)	0.002
***IDH1 R132* mutation (Positive vs. Negative)**	149	0.27(0.06–0.92)	0.052
***IDH1 R140* mutation (Positive vs. Negative)**	149	2.03(0.61–7.89)	0.266
***RAS* mutation (Positive vs. Negative)**	150	1.67(0.39–8.37)	0.495
***NPM1* mutation (Positive vs. Negative)**	150	0.34(0.14–0.75)	0.01
***DNMT3A* mutation (Positive vs. Negative)**	109	2.09(0.73–6.51)	0.179
***RUNX1* mutation (Positive vs. Negative)**	109	0.30(0.06–1.08)	0.085

### High *COMMD7* impacted the prognosis of AML in patients with different clinicopathological status

**Figure 8:**
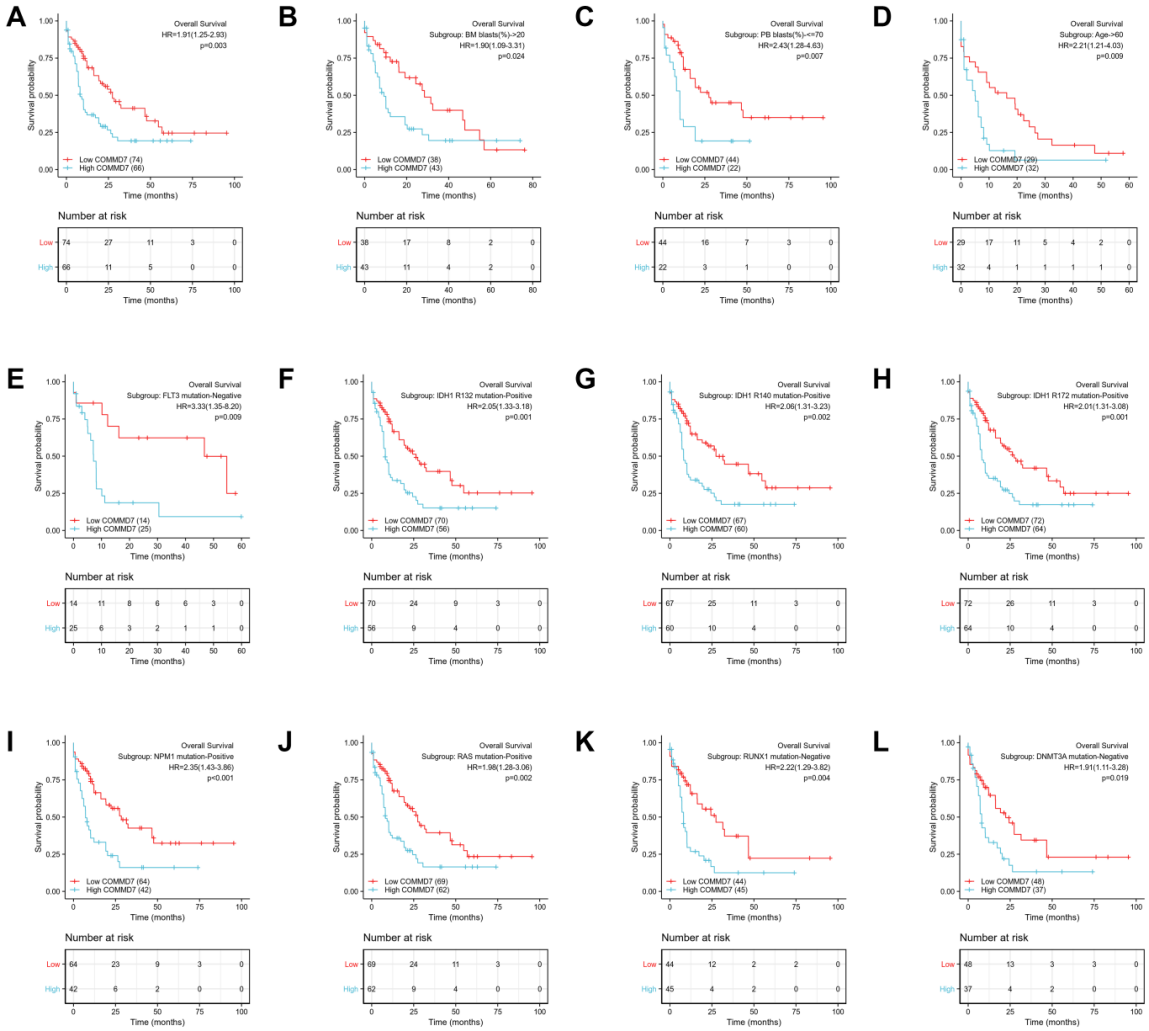
**High expression of *COMMD7* was associated with poor OS in AML patients.** (**A**) Kaplan-Meier curves in all AML patients. (**B**) Kaplan-Meier curves in AML patients with BM blasts > 20%. (**C**) Kaplan-Meier curves in AML patients with PB blasts ≤ 70%. (**D**) Kaplan-Meier curves in AML patients with age ≥ 60. (**E**–**L**) Kaplan-Meier curves in subgroups with *FLT3* mutation-negative, IDH1 R132 mutation-positive, IDH1 R140 mutation-positive, R172 mutation-positive, NPM1 mutation-positive, RAS mutation-positive, RUX1 mutation-negative, and DNMT3A mutation-negative in AML patients.

The relationship between *COMMD7* expression and prognosis was analyzed in AML patients by using Kaplan-Meier. As seen in Figure 8A, patients with high expression of *COMMD7* had a strongly worse prognosis than those with low *COMMD7* expression (hazard ratio [HR], 1.91(1.25-2.93); *P* = 0.003). Kaplan-Meier analysis presented that high expressed *COMMD7* correlated with poor prognosis in the subgroups of BM blasts≥ 20% (*P* = 0.024), PB blasts ≤ 70% (*P* = 0.007), age >60 (*P* = 0.009), *FLT3* mutation negative (*P* = 0.009), *IDH1 R132* mutation positive (*P* = 0.001), *R140* mutation positive (*P* = 0.002), *R172* mutation positive (*P* = 0.001), *NPM1* mutation positive (*P* < 0.001), *RAS* mutation positive (*P* = 0.002), *RUX1* mutation negative (*P* = 0.004), and *DNMT3A* mutation negative (*P* = 0.019) (Figure 8B–8L).

**Figure 9:**
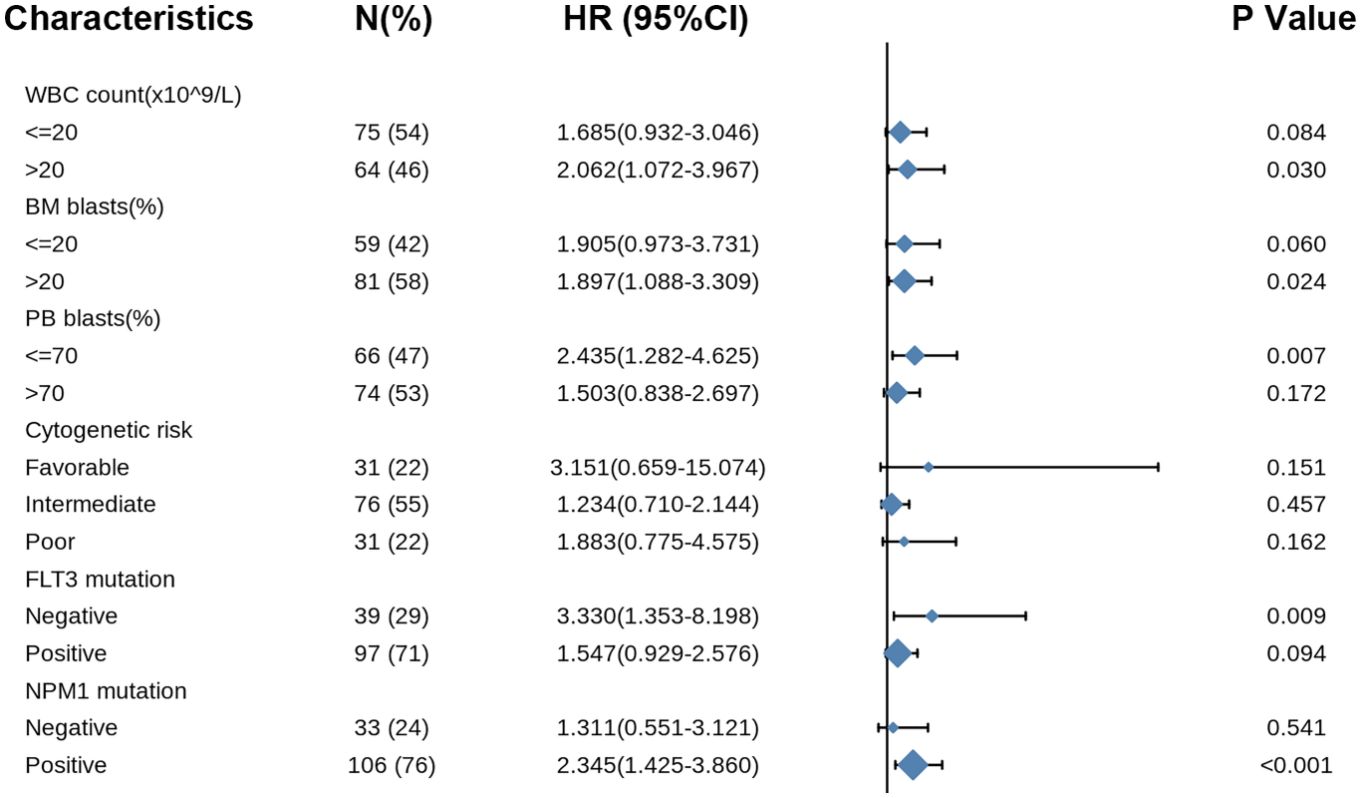
**Forest plot showed that *COMMD7* predicted poor prognosis in the subgroup of WBC count** (>20 × 10^9^/L) (HR = 2.062, *P* = 0.030), BM blasts (>20%) (HR = 1.897, *P* = 0.024), PB blasts (>70%) (HR = 2.435, *P* = 0.007), *FLT3* mutation negative (HR = 3.330, *p* = 0.009), and *NPM1* mutation positive (HR = 2.345, *P* < 0.001).

Likewise, the forest plot illustrated the prognostic value of *COMMD7* in various AML subtypes using univariate Cox regression, with a conclusion consistent with the above results (Figure 9).

Hereafter, univariate Cox proportional hazards regression was used to assess the factors influencing OS, disclosing that *COMMD7* (high- vs. low-, *P* = 0.003) was a predictive factor for worse OS, so did cytogenetic risk (poor & intermediate vs. favorable, *P* < 0.001) and age (>60 vs. ≤60, *P* < 0.001) (Table 3). Cytogenetic risk, age and *COMMD7* were then included in multivariate Cox regression, suggesting that age > 60 (*P* < 0.001) and high expression of *COMMD7* (*P* = 0.01) were independent prognostic factors for worse OS (*P* < 0.05).

**Table 3:** Univariate and multivariate Cox’s regression analysis of factors associated with OS in AML.

**Characteristics**	**HR (95% CI)** **Univariate analysis**	***P* value** **Univariate analysis**	**HR (95% CI)** **Multivariate analysis**	***P* value** **Multivariate analysis**
**WBC count** **(x10^9^/L)** **(>20 vs. <=20)**	1.161 (0.760–1.772)	0.49		
**PB blasts (%)** **(>70 vs. <=70)**	1.230 (0.806–1.878)	0.338		
**BM blasts(%)** **(>20 vs. <=20)**	1.165 (0.758–1.790)	0.486		
**Cytogenetic risk** **(Favorable vs. Poor&Intermediate)**	0.312 (0.160–0.606)	<0.001	0.535 (0.261–1.097)	0.088
**Gender** **(Male vs. Female)**	1.030 (0.674–1.572)	0.892		
**Age** **(>60 vs. <=60)**	3.333 (2.164–5.134)	<0.001	3.374 (2.130–5.344)	<0.001
**Race (White vs. Asian&Black** **or African American)**	1.200 (0.485–2.966)	0.693		
***FLT3* mutation** **(Positive vs. Negative)**	0.787 (0.496–1.248)	0.309		
***IDH1 R132* mutation** **(Positive vs. Negative)**	1.702 (0.689–4.205)	0.249		
***IDH1 R140* mutation** **(Positive vs. Negative)**	0.884 (0.442–1.769)	0.727		
***IDH1 R172* mutation** **(Positive vs. Negative)**	1.641 (0.228–11.804)	0.623		
***RAS* mutation** **(Positive vs. Negative)**	1.555 (0.568–4.254)	0.39		
***NPM1* mutation** **(Positive vs. Negative)**	0.879 (0.546–1.416)	0.596		
***DNMT3A* mutation** **(Positive vs. Negative)**	1.404 (0.731–2.696)	0.308		
***RUNX1* mutation** **(Positive vs. Negative)**	1.119 (0.553–2.267)	0.754		
***COMMD7*** **(High vs. Low)**	1.914 (1.251–2.927)	0.003	1.850 (1.158–2.954)	0.01

### Prognostic model of *COMMD7* in AML

**Figure 10:**
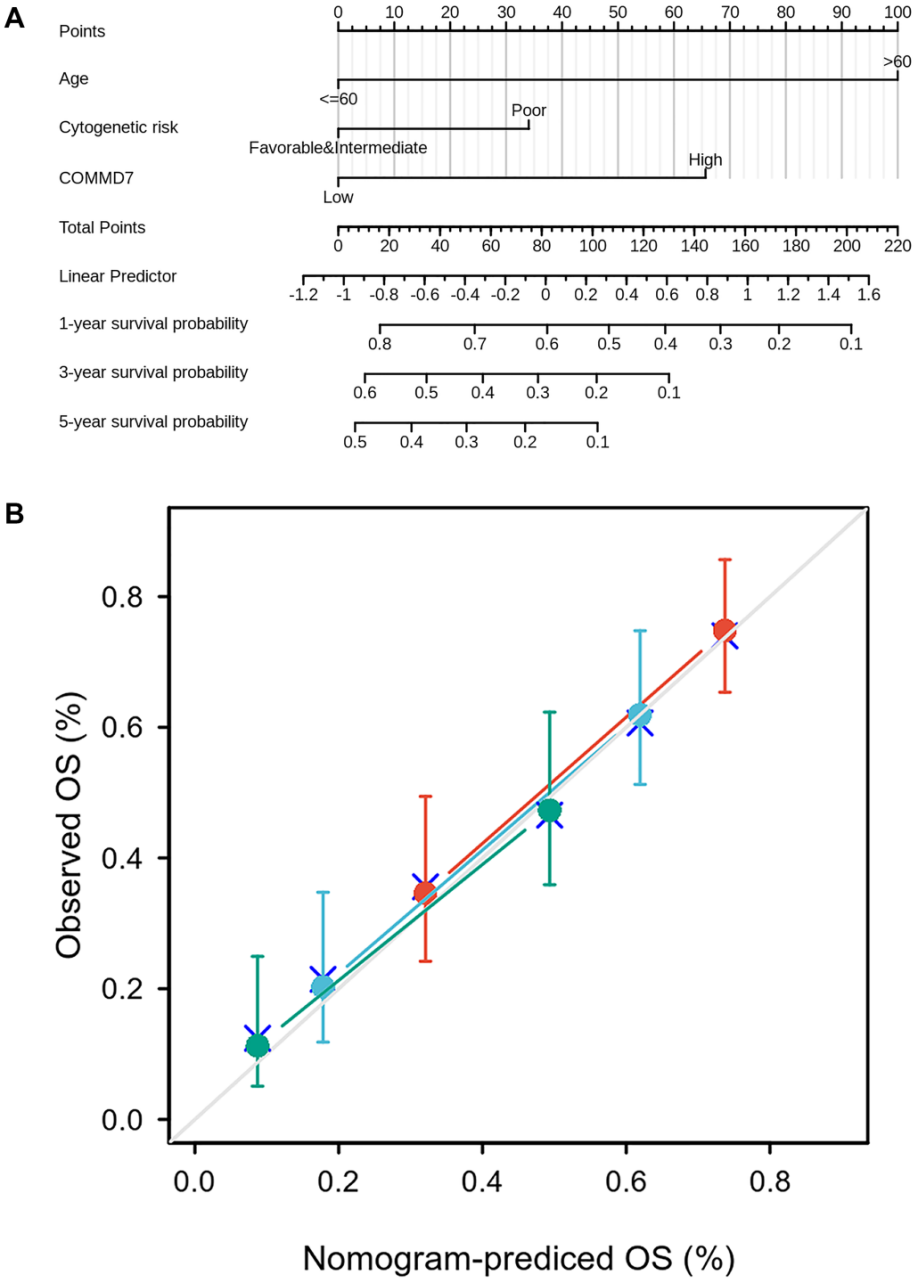
**A prognostic predictive model of *COMMD7* in AML.** (**A**) Nomogram for predicting the probability of 1-, 3-, 5-year OS for AML. (**B**) Calibration plot of the nomogram for predicting the probability of OS at 1, 3, and 5 years.

To better predict AML patients' prognosis, a nomogram was constructed based on the Cox regression analysis results using the RMS R package (Figure 10A). Three independent prognostic factor variables, age, cytogenetic risk, and *COMMD7* expression, were included in the model, selected into the prediction model at a statistical significance level of 0.2. Based on multivariate Cox analysis, a point scale was used to assign points to these variables. The straight line was drawn upward to determine the points of the variables, and the sum of the points assigned to each variable was rescaled to a range of 0–100. The points of each variable were accumulated and recorded as the total points. The probability of AML patient survival at 1-, 3-, and 5-year was determined by drawing a line from the total point axis straight down to the outcome axis. The 1-year survival probability was determined by drawing a vertical line downward on the total point axis along the 162-direction ending axis, suggesting the probability of 1-year survival < 20%, both of the probability of 3- and 5-year < 10%. The prediction results of the nomogram calibration curve of OS were consistent with all patients' observation results (Figure 10B).

## DISCUSSION

*COMMD7* is a member of the COMMD family defined by the presence of a conserved and unique motif termed the COMM (copper metabolism gene MURR1) domain, which functions as an interface for protein-protein interactions [4]. Several studies had revealed that *COMMD7* was involved in the regulation of NF-kappa B signaling [5, 8]. It was known that un-correct regulation of the NF-kB pathway had been linked to various tumors. Studies in several tumors such as hepatocellular carcinoma and pancreatic ductal adenocarcinoma have evaluated the expression and function of *COMMD7* in tumor development [5, 6]. However, little is known about the expression of *COMMD7* and prognostic value in AML.

The present study's central result was that high-expressed *COMMD7* in AML was associated with high BM/PB blasts, intermediate-high cytogenetic risk, and poor prognosis. Via GSEA gene enrichment analysis, low-expressed *COMMD7* was associated with *NPM1* mutation, *PML-RARa* fusion, *AML-ETO* fusion, and *CBFB-MYH11* fusion, which are excellent prognostic factors. In contrast, high-expressed *COMMD7* was associated with Wnt, RAS, MAPK, and Hedgehog pathways, suggesting that *COMMD7* was not only a potential prognostic biomarker but also a promising therapeutic target by affecting oncogenesis-related pathways in AML.

It is worth noting that the most clinically relevant finding was that high expression of *COMMD7* was associated with poor survival. Multivariate Cox regression analysis showed that high expression of *COMMD7* was another independent prognostic factor besides age (>60 years). The establishment of the nomogram prediction model further confirmed the predictive effect of *COMMD7* expression on prognosis. Therefore, *COMMD7* may serve as a new adverse prognostic factor in AML patients.

More importantly, it was found that high expression of *COMMD7* predicted poor prognosis in a subgroup of AML patients with *NPM1* mutation. *NPM1* mutation occurs in about 30% of newly diagnosed AML, which is to date one of the most frequent genetic alterations identified in AML. Isolated *NPM1* mutations are generally considered to have a positive prognostic effect on AML [21, 22]. Unfortunately, approximately 30-70% of AML patients with *NPM1* mutations relapse within five years, with age and *FLT3-ITD* mutations reported to be influencing factors [23, 24]. The relevance of cooperation between *NPM1* and other mutations with different outcomes in driving AML has been reported in several other studies [21, 22, 25, 26]. In view that the pivotal elements and mechanisms are still not completely clear, herein, we discovered that AML patients with high expression of *COMMD7* leading to *NPM1* mutations had a poor prognosis. Further research is required to verify the effect of high COMMD7 expression on AML with NPM1-mutation and explore its underlying mechanism.

In addition, Wnt, RAS, MAPK, and Hedgehog pathways were found to be closely relevant with high expression of *COMMD7* in AML. Wnt signaling was convinced to be up-regulated through a variety of mechanisms in AML that are necessary for the maintenance of leukemia stem cells [27, 28]. A high incidence of gene mutations in RAS/MAPK pathway was identified in AML. Hedgehog pathway was related to AML cell resistance to drugs and radiotherapy, resulting in poor prognosis in AML patients. Here, *COMMD7* was found to be associated with these pathways and may be involved in the genesis and maintenance of leukemia cells, calling for further studies to confirm our results and explore the specific regulatory mechanism of *COMMD7* as well as these pathways.

In immune cell infiltration analysis, high expression of *COMMD7* was associated with higher CD56(bright) NK cells. Human NK cells account for 10–15% of circulating lymphocytes, of which CD56(bright) and CD56(dim) NK cells are the primary two subsets. CD56(bright) NK cells have been considered as immature NK cells, precursors of CD56(dim) NK cells. Compared with CD56(dim) NK cells, CD56(bright) NK cells are characterized by high cytokine production and low cytotoxic capacity [29–31]. Notably, NK cells play a “double-edged sword” role in the generation of tumors. Traditionally, NK cells have been considered to play an important role in immunosurveillance and thus have antitumor effects [32]. Recently, a series of studies have shown that CD56(bright) NK cells promote tumor development [31, 33–35]. CD56(bright) NK cell infiltration increased in lung cancer, colorectal cancer, breast cancer, et al. [33, 34, 36, 37]. Promoting tumor angiogenesis, tumor immune escape, and loss of activity to kill tumor stem cells have been demonstrated to be involved in promoting the malignant progression of CD56(bright) NK cells [33, 38, 39]. Cytokines in the tumor microenvironment play a regulatory role in promoting CD56(bright) NK cell tumors [39, 40]. In this study, NK CD56(bright) cells' infiltration was positively correlated with *COMMD7* expression. Through Kaplan-Meier survival analysis, high *COMMD7* expression was found to be associated with poor prognosis in AML patients. AML blast cells have been reported to evade NK cell immunosurveillance by diminishing the expression of several activated receptors [29]. However, the relationship between CD56(bright) NK cells and AML has not been fully elaborated. Hence, according to our findings and the above research reports, the relationship between *COMMD7* and CD56(bright) and CD56(dim) NK cells, and whether *COMMD7* and CD56(bright) NK cells are involved in the immune escape in AML still deserve further exploration.

Moreover, Cox analysis in the present study indicated that *COMMD7* might have the ability to become an independent predictor of poor prognosis in AML after adjusting for routine clinical features. Multivariate Cox regression analysis showed that age>60 years and high *COMMD7* expression were the independent prognostic factors for OS deterioration. A nomogram prognosis map was constructed by combining COMMD7 with cytogenetic risk and age to obtain a more accurate prognosis prediction model. The C-index *COMMD7*-related Cox model predicted the OS to be 0.754 (0.728–0.780). The calibration chart showed optimal agreement between the predictions of the nomogram associated with *COMMD7* and the actual observations of 1-year, 3-year, and 5-year OS probabilities. As previously reported, older age (>60), high cytogenetic risk, high WBC count (>20 × 10^9^/L), *FLT3* mutation-positive, and *NPM1* mutation-negative were independent factors predicting poor prognosis of AML [1]. According to the Cox analysis and nomogram model, it seems that *COMMD7* may potentially have better predictive power than cytogenetic risk, WBC count, *FLT3* mutation status, and *NPM1* mutation status. From this point of view, our model may provide a personalized score for individual AML patients.

However, the limitation of this study lies in the small sample size. Also, to ensure greater reliability and representativeness of the findings and assumptions, the sample should be expanded for further research in the future. Clinical samples should be used to verify the prognostic predictive role of *COMMD7* mRNA and protein in AML. Experimental validation should also be performed to investigate the regulatory mechanisms between *COMMD7* and the genetic alterations (such as NPM1 and FLT3) and essential pathways selected by GSEA analysis. A mass of plans has been formulated for some recent laboratory work.

## CONCLUSIONS

In summary, this study disclosed for the first time that *COMMD7* expression increased in AML, which is also related to poor prognosis. Moreover, Wnt, RAS, MAPK, and Hedgehog pathways may be the essential pathways participating in the regulation of *COMMD7* in AML. More intriguingly, *COMMD7* may reverse the *NPM1* mutation from a good role in AML. Further verification should be carried out to reveal the biological impacts of *COMMD7* in AML.

## Supplementary Material

Supplementary Figure 1

Supplementary Table 1

Supplementary Table 2

Supplementary Table 3

Supplementary Table 4

## References

[1] Papaemmanuil E, Gerstung M, Bullinger L, Gaidzik VI, Paschka P, Roberts ND, Potter NE, Heuser M, Thol F, Bolli N, Gundem G, Van Loo P, Martincorena I, et al. Genomic Classification and Prognosis in Acute Myeloid Leukemia. N Engl J Med. 2016; 374:2209–21. 10.1056/NEJMoa1516192 27276561 PMC4979995

[2] Lindsley RC, Mar BG, Mazzola E, Grauman PV, Shareef S, Allen SL, Pigneux A, Wetzler M, Stuart RK, Erba HP, Damon LE, Powell BL, Lindeman N, et al. Acute myeloid leukemia ontogeny is defined by distinct somatic mutations. Blood. 2015; 125:1367–76. 10.1182/blood-2014-11-610543 25550361 PMC4342352

[3] Döhner H, Estey E, Grimwade D, Amadori S, Appelbaum FR, Büchner T, Dombret H, Ebert BL, Fenaux P, Larson RA, Levine RL, Lo-Coco F, Naoe T, et al. Diagnosis and management of AML in adults: 2017 ELN recommendations from an international expert panel. Blood. 2017; 129:424–47. 10.1182/blood-2016-08-733196 27895058 PMC5291965

[4] Burstein E, Hoberg JE, Wilkinson AS, Rumble JM, Csomos RA, Komarck CM, Maine GN, Wilkinson JC, Mayo MW, Duckett CS. COMMD proteins, a novel family of structural and functional homologs of MURR1. J Biol Chem. 2005; 280:22222–32. 10.1074/jbc.M501928200 15799966

[5] You N, Li J, Gong Z, Huang X, Wang W, Wang L, Wu K, Zheng L. COMMD7 functions as molecular target in pancreatic ductal adenocarcinoma. Mol Carcinog. 2017; 56:607–24. 10.1002/mc.22520 27350032

[6] You N, Li J, Huang X, Wu K, Tang Y, Wang L, Li H, Mi N, Zheng L. COMMD7 promotes hepatocellular carcinoma through regulating CXCL10. Biomed Pharmacother. 2017; 88:653–57. 10.1016/j.biopha.2017.01.046 28142122

[7] Esposito E, Napolitano G, Pescatore A, Calculli G, Incoronato MR, Leonardi A, Ursini MV. COMMD7 as a novel NEMO interacting protein involved in the termination of NF-κB signaling. J Cell Physiol. 2016; 231:152–61. 10.1002/jcp.25066 26060140

[8] Zheng L, You N, Huang X, Gu H, Wu K, Mi N, Li J. COMMD7 Regulates NF-κB Signaling Pathway in Hepatocellular Carcinoma Stem-like Cells. Mol Ther Oncolytics. 2018; 12:112–23. 10.1016/j.omto.2018.12.006 30719501 PMC6350112

[9] Zheng L, Deng CL, Wang L, Huang XB, You N, Tang YC, Wu K, Liang P, Mi N, Li J. COMMD7 is correlated with a novel NF-κB positive feedback loop in hepatocellular carcinoma. Oncotarget. 2016; 7:32774–84. 10.18632/oncotarget.9047 27129158 PMC5078050

[10] Vivian J, Rao AA, Nothaft FA, Ketchum C, Armstrong J, Novak A, Pfeil J, Narkizian J, Deran AD, Musselman-Brown A, Schmidt H, Amstutz P, Craft B, et al. Toil enables reproducible, open source, big biomedical data analyses. Nat Biotechnol. 2017; 35:314–16. 10.1038/nbt.3772 28398314 PMC5546205

[11] Goldman MJ, Craft B, Hastie M, Repečka K, McDade F, Kamath A, Banerjee A, Luo Y, Rogers D, Brooks AN, Zhu J, Haussler D. Visualizing and interpreting cancer genomics data via the Xena platform. Nat Biotechnol. 2020; 38:675–78. 10.1038/s41587-020-0546-8 32444850 PMC7386072

[12] Li K, Luo H, Luo H, Zhu X. Clinical and prognostic pan-cancer analysis of m6A RNA methylation regulators in four types of endocrine system tumors. Aging (Albany NY). 2020; 12:23931–44. 10.18632/aging.104064 33237039 PMC7762517

[13] Wang JD, Zhou HS, Tu XX, He Y, Liu QF, Liu Q, Long ZJ. Prediction of competing endogenous RNA coexpression network as prognostic markers in AML. Aging (Albany NY). 2019; 11:3333–47. 10.18632/aging.101985 31164492 PMC6555472

[14] Love MI, Huber W, Anders S. Moderated estimation of fold change and dispersion for RNA-seq data with DESeq2. Genome Biol. 2014; 15:550. 10.1186/s13059-014-0550-8 25516281 PMC4302049

[15] Yu G, Wang LG, Han Y, He QY. clusterProfiler: an R package for comparing biological themes among gene clusters. OMICS. 2012; 16:284–87. 10.1089/omi.2011.0118 22455463 PMC3339379

[16] Bindea G, Mlecnik B, Tosolini M, Kirilovsky A, Waldner M, Obenauf AC, Angell H, Fredriksen T, Lafontaine L, Berger A, Bruneval P, Fridman WH, Becker C, et al. Spatiotemporal dynamics of intratumoral immune cells reveal the immune landscape in human cancer. Immunity. 2013; 39:782–95. 10.1016/j.immuni.2013.10.003 24138885

[17] Szklarczyk D, Gable AL, Lyon D, Junge A, Wyder S, Huerta-Cepas J, Simonovic M, Doncheva NT, Morris JH, Bork P, Jensen LJ, Mering CV. STRING v11: protein-protein association networks with increased coverage, supporting functional discovery in genome-wide experimental datasets. Nucleic Acids Res. 2019; 47:D607–D613. 10.1093/nar/gky1131 30476243 PMC6323986

[18] Demchak B, Hull T, Reich M, Liefeld T, Smoot M, Ideker T, Mesirov JP. Cytoscape: the network visualization tool for GenomeSpace workflows. F1000Res. 2014; 3:151. 10.12688/f1000research.4492.2 25165537 PMC4133763

[19] Bandettini WP, Kellman P, Mancini C, Booker OJ, Vasu S, Leung SW, Wilson JR, Shanbhag SM, Chen MY, Arai AE. MultiContrast Delayed Enhancement (MCODE) improves detection of subendocardial myocardial infarction by late gadolinium enhancement cardiovascular magnetic resonance: a clinical validation study. J Cardiovasc Magn Reson. 2012; 14:83. 10.1186/1532-429X-14-83 23199362 PMC3552709

[20] Isidro-Sánchez J, Akdemir D, Montilla-Bascón G. Genome-Wide Association Analysis Using R. Methods Mol Biol. 2017; 1536:189–207. 10.1007/978-1-4939-6682-0_14 28132152

[21] Falini B, Brunetti L, Sportoletti P, Martelli MP. NPM1-mutated acute myeloid leukemia: from bench to bedside. Blood. 2020; 136:1707–21. 10.1182/blood.2019004226 32609823

[22] Kunchala P, Kuravi S, Jensen R, McGuirk J, Balusu R. When the good go bad: Mutant NPM1 in acute myeloid leukemia. Blood Rev. 2018; 32:167–83. 10.1016/j.blre.2017.11.001 29157973

[23] Patel SS, Kuo FC, Gibson CJ, Steensma DP, Soiffer RJ, Alyea EP 3rd, Chen YA, Fathi AT, Graubert TA, Brunner AM, Wadleigh M, Stone RM, DeAngelo DJ, et al. High *NPM1*-mutant allele burden at diagnosis predicts unfavorable outcomes in de novo AML. Blood. 2018; 131:2816–25. 10.1182/blood-2018-01-828467 29724895 PMC6265642

[24] Webersinke G, Kranewitter W, Deutschbauer S, Zach O, Hasenschwandtner S, Wiesinger K, Erdel M, Marschon R, Böhm A, Tschurtschenthaler G. Switch of the mutation type of the NPM1 gene in acute myeloid leukemia (AML): relapse or secondary AML? Blood Cancer J. 2014; 4:e221. 10.1038/bcj.2014.42 24972150 PMC4080213

[25] Pepper M, Tan B. Acute myeloid leukemia with NPM1 and FLT3 ITD mimicking acute promyelocytic leukemia. Blood. 2020; 136:1467. 10.1182/blood.2020007198 32941638

[26] Cocciardi S, Dolnik A, Kapp-Schwoerer S, Rücker FG, Lux S, Blätte TJ, Skambraks S, Krönke J, Heidel FH, Schnöder TM, Corbacioglu A, Gaidzik VI, Paschka P, et al. Clonal evolution patterns in acute myeloid leukemia with NPM1 mutation. Nat Commun. 2019; 10:2031. 10.1038/s41467-019-09745-2 31048683 PMC6497712

[27] Gruszka AM, Valli D, Alcalay M. Wnt Signalling in Acute Myeloid Leukaemia. Cells. 2019; 8:1403. 10.3390/cells8111403 31703382 PMC6912424

[28] Simon M, Grandage VL, Linch DC, Khwaja A. Constitutive activation of the Wnt/beta-catenin signalling pathway in acute myeloid leukaemia. Oncogene. 2005; 24:2410–20. 10.1038/sj.onc.1208431 15735743

[29] Sanchez-Correa B, Campos C, Pera A, Bergua JM, Arcos MJ, Bañas H, Casado JG, Morgado S, Duran E, Solana R, Tarazona R. Natural killer cell immunosenescence in acute myeloid leukaemia patients: new targets for immunotherapeutic strategies? Cancer Immunol Immunother. 2016; 65:453–63. 10.1007/s00262-015-1720-6 26059279 PMC11029066

[30] Montaldo E, Vacca P, Moretta L, Mingari MC. Development of human natural killer cells and other innate lymphoid cells. Semin Immunol. 2014; 26:107–13. 10.1016/j.smim.2014.01.006 24559836

[31] Michel T, Poli A, Cuapio A, Briquemont B, Iserentant G, Ollert M, Zimmer J. Human CD56bright NK Cells: An Update. J Immunol. 2016; 196:2923–31. 10.4049/jimmunol.1502570 26994304

[32] Klein E, Vánky F, Vose BM. Natural killer and tumor recognizing lymphocyte activity in tumor patients. Haematologia (Budap). 1978-1979; 12:107–12. 395038

[33] Bruno A, Focaccetti C, Pagani A, Imperatori AS, Spagnoletti M, Rotolo N, Cantelmo AR, Franzi F, Capella C, Ferlazzo G, Mortara L, Albini A, Noonan DM. The proangiogenic phenotype of natural killer cells in patients with non-small cell lung cancer. Neoplasia. 2013; 15:133–42. 10.1593/neo.121758 23441128 PMC3579316

[34] Bruno A, Bassani B, D'Urso DG, Pitaku I, Cassinotti E, Pelosi G, Boni L, Dominioni L, Noonan DM, Mortara L, Albini A. Angiogenin and the MMP9-TIMP2 axis are up-regulated in proangiogenic, decidual NK-like cells from patients with colorectal cancer. FASEB J. 2018; 32:5365–77. 10.1096/fj.201701103R 29763380

[35] Poli A, Michel T, Thérésine M, Andrès E, Hentges F, Zimmer J. CD56bright natural killer (NK) cells: an important NK cell subset. Immunology. 2009; 126:458–65. 10.1111/j.1365-2567.2008.03027.x 19278419 PMC2673358

[36] Rocca YS, Roberti MP, Arriaga JM, Amat M, Bruno L, Pampena MB, Huertas E, Loria FS, Pairola A, Bianchini M, Mordoh J, Levy EM. Altered phenotype in peripheral blood and tumor-associated NK cells from colorectal cancer patients. Innate Immun. 2013; 19:76–85. 10.1177/1753425912453187 22781631

[37] Mamessier E, Sylvain A, Thibult ML, Houvenaeghel G, Jacquemier J, Castellano R, Gonçalves A, André P, Romagné F, Thibault G, Viens P, Birnbaum D, Bertucci F, et al. Human breast cancer cells enhance self tolerance by promoting evasion from NK cell antitumor immunity. J Clin Invest. 2011; 121:3609–22. 10.1172/JCI45816 21841316 PMC3171102

[38] Bhatia A, Kumar Y. Cellular and molecular mechanisms in cancer immune escape: a comprehensive review. Expert Rev Clin Immunol. 2014; 10:41–62. 10.1586/1744666X.2014.865519 24325346

[39] Konjević GM, Vuletić AM, Mirjačić Martinović KM, Larsen AK, Jurišić VB. The role of cytokines in the regulation of NK cells in the tumor environment. Cytokine. 2019; 117:30–40. 10.1016/j.cyto.2019.02.001 30784898

[40] Maghazachi AA. Role of chemokines in the biology of natural killer cells. Curr Top Microbiol Immunol. 2010; 341:37–58. 10.1007/82_2010_20 20369317

