# Supplementary material for: High expression of COMMD7 is an adverse prognostic factor in acute myeloid leukemia
Source: Aging (Albany NY). 2021 Apr 23;13(8):11988–2006. doi: 10.18632/aging.202901 (PMC8109082; doi:10.18632/aging.202901)
Supplement: Supplementary Figure 1 [file aging-13-202901-s001.pdf]

## SUPPLEMENTARY FIGURE

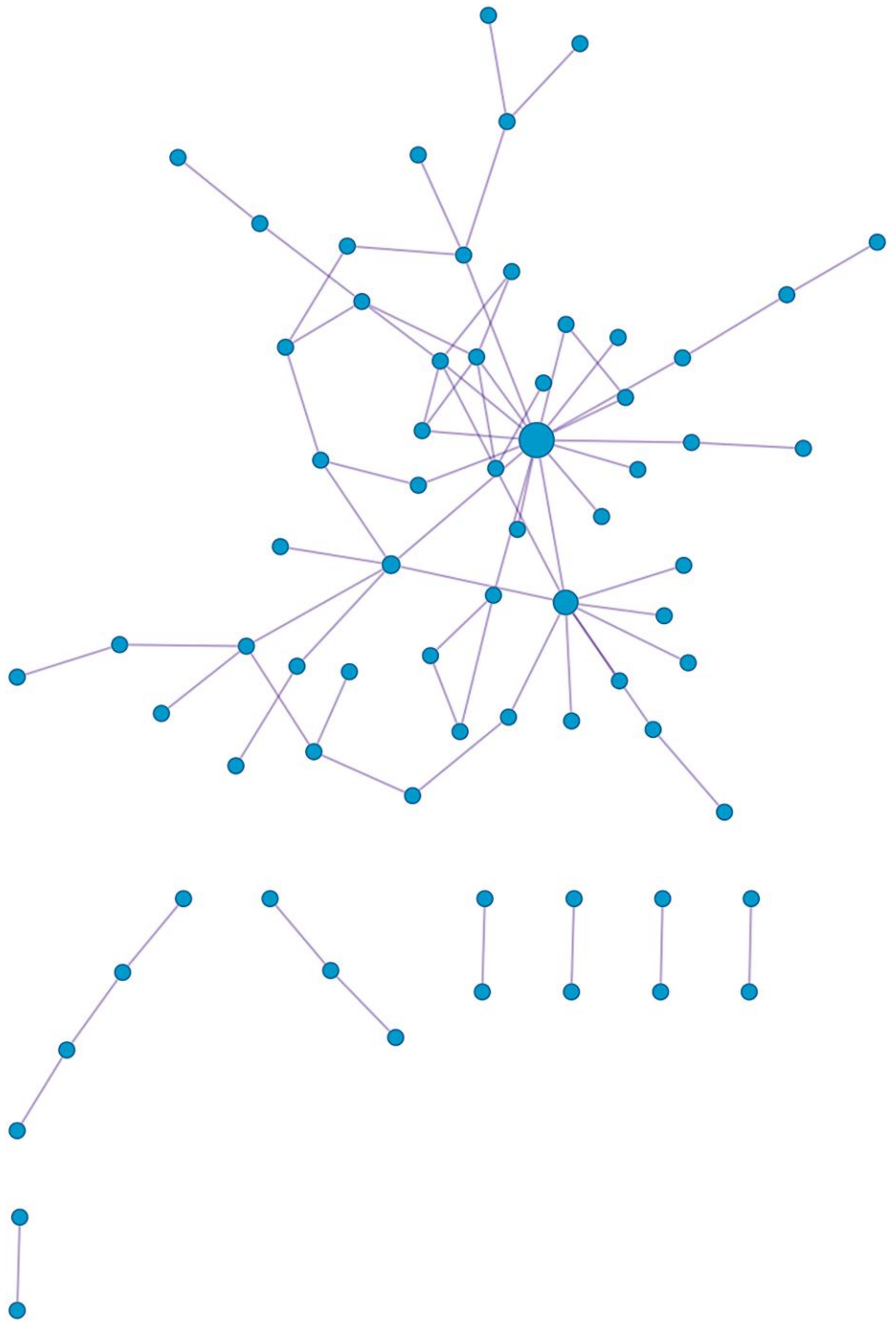

**Supplementary Figure 1. Protein-protein interaction network from Metascape.**
