# Supplementary material for: High expression of COMMD7 is an adverse prognostic factor in acute myeloid leukemia
Source: Aging (Albany NY). 2021 Apr 23;13(8):11988–2006. doi: 10.18632/aging.202901 (PMC8109082; doi:10.18632/aging.202901)
Supplement: Supplementary Table 4 [file aging-13-202901-s005.pdf]

SUPPLEMENTARY TABLE

Supplementary Table 4. Pathway and process enrichment analysis of Protein-protein interaction network and MCODE from Metascape.

GO	Description	Log10 (*P*)
**R-HSA-9675108**	Nervous system development	–13.3
**R-HSA-9006934**	Signaling by Receptor Tyrosine Kinases	–13.0
**GO:0007169**	transmembrane receptor protein tyrosine kinase signaling pathway	–12.6
